# Effects of the Interactive Use of Performance Measurement Systems on Job Performance: Mediation Effect of Organizational Learning

**DOI:** 10.3389/fpsyg.2019.03059

**Published:** 2020-01-31

**Authors:** Lu Zhang, Wenjun Yu

**Affiliations:** ^1^Business School, Ningbo University, Ningbo, China; ^2^Academy of Neuroeconomics and Neuromanagement, Ningbo University, Ningbo, China

**Keywords:** performance measurement system, interactive performance measurement system use, organizational learning, resource-based view, job performance, structural equation model

## Abstract

Interactive controls that focus on communication and continuous learning are very important to achieve a competitive advantage. To better understand the underlying mechanism of how performance measurement systems (PMSs) improve job performance, a mediation model was constructed in the current study to examine organizational learning as a possible mediating variable. Data were collected using a questionnaire in China with a sample size of 191 managers. Structural equation model and Smart-PLS methods were used to test the hypotheses. Results yielded significant direct effects between the interactive use of PMS on organizational learning and job performance. Additionally, organizational learning was found to play a mediating role in the relationship between interactive use of PMS and job performance. These findings highlight the importance of interactive PMS use, as well as the underlying mechanisms among PMS, organizational learning, and job performance, and further help the management clarify how organizational learning affects performance, providing a framework for building a sustainable competitive advantage.

## Introduction

The current business climate includes global competition, high market uncertainty, diverse customer needs, and rapidly emerging technology. In such an environment, a controlled system with a strong financial orientation is no longer appropriate and can even be counterproductive. In order to maintain a sustainable competitive advantage, managers need to stimulate their employees to learn by continuously evaluating their performance and providing information about market demands, technologies, and resources (Hurley and Hult, 1998; Mu and Di Benedetto, 2011).

Performance measurement systems (PMSs) are formalized systems that use metrics to plan, report, and monitor procedures in an organization (Henri, 2006). PMSs facilitate the connection to learning and improvement, as well as defines triggers for change (Bourne, 2005; Cestari et al., 2018). These systems utilize both financial and non-financial measures to monitor performance (Ittner et al., 2003; Speklé and Verbeeten, 2014; Cäker and Siverbo, 2018).

Generally, PMSs are used for two different purposes: first, as a diagnostic capacity, which reflects the traditional feedback role of a PMS to support the implementation of strategy; and second, in an interactive role, which is associated with information disseminated throughout an organization focusing on learning, stimulating communication, and guiding the emergence of new strategies for a dynamic environment. The diagnostic use evaluates business performance of managers by various assessment indicators, whereas interactive use attaches importance to the future, indicates necessary changes, and emphasizes effective communication throughout the organization (Simons, 1995; Henri, 2006).

The turbulent business environment highlights the importance of interactive PMS use, as it plays a critical role in promoting employee discussion, which facilitates learning (Simons, 1995). Interactive applications are not used to observe workers, enforce conformity, and/or exert control, but it is more of a learning behavior-driven process. The resource-based view (RBV) is built on the principle that competitiveness is a function of specific internal resources and capabilities possessed by a firm (Lengnick-Hall and Wolff, 1999; Henri, 2006). This requires organizations to have a strong learning capacity, namely, organizational learning. According to Fiol and Lyles (1985), organizational learning is “the development of insights, knowledge, and associations among past actions, the effectiveness of these actions, and future actions.” If an organization wishes to improve operations, it should strive to continuously enhance the knowledge accumulation of employees, preferably by enhancing learning capability. Specifically, through an interactive use mechanism, organizations facilitate discussions among employees, fostering an inspirational and participative atmosphere (Schäffer et al., 2014), thus increasing overall organizational learning and further improving job performance.

Our objective is to explain the mechanism by which interactive PMS is presumed to affect organizational learning and job performance. When designing an effective PMS, underlying mechanisms should be studied. However, the operations management literature is currently limited to the formal design of PMSs and contains little information concerning PMS usage from a RBV theory standpoint (e.g., Chenhall, 2005; Henri, 2006; Widener, 2007; Koufteros et al., 2014). Pavlov and Bourne (2011) have stated that the relationship between PMS and performance is poorly understood. For instance, prior research notes that it has not been demonstrated exactly how PMSs are linked to performance (Ittner et al., 2003) and that the effects may actually be reflected by capabilities (Henri, 2006; Franco-Santos et al., 2012). In other words, PMSs must be aligned with the specific capabilities of workers to become a competitive advantage. Furthermore, there is still a lack of comprehension on how interactive PMS use impacts employee performance at the individual level. Performance category can be complemented differently than the existing empirical literature (Henri, 2006), thereby facilitating the understanding of this phenomenon. Research in this area is important to progress the interactive PMS field and to support evidence-based management initiatives. Hence, three specific research questions were investigated in this study: (i) Does the implementation of interactive PMS benefit employee’s job performance? (ii) Does the use of interactive PMS contribute to an improvement in organizational learning? (iii) How does the interactive use of PMS actually affect employee job performance through organizational learning? A theoretical model was tested with empirical data gathered from a survey conducted in the Beijing, Shanghai, Zhejiang, and Jiangsu regions of China, which is one of the world’s most successful emerging economies.

## Theoretical Background and Hypotheses

### Interactive Performance Measurement Systems and Organizational Learning

A PMS, defined as a mechanism to allocate responsibilities and decision rights, sets performance targets and rewards the achievement of targets (Otley, 1999; Lee and Yang, 2011). Interactive PMS use is defined as a measurement system that is used to focus attention on the constantly changing information that top-level managers consider to be of strategic importance (Bisbe and Otley, 2004). According to Simons (1995), when a PMS is used interactively, the information generated is an important and recurring agenda for senior management, frequent and regular attention is fostered throughout the organization, data are interpreted and discussed among managers at all levels of the organization, and “continual challenge and debate occur concerning underlying data, assumptions, and action plans” (Simons, 1995, p. 97). Using a PMS interactively improves communication, stimulates the development of new ideas and initiatives, and helps to adapt to competitive environments (Widener, 2007; Koufteros et al., 2014). Consequently, interactive PMS supports opportunity seeking, creativity, dialog, and learning and demands constant and intense managerial attention (Schäffer et al., 2014).

Previous research suggests that interactive use of PMS has a positive impact on organizational learning. Argyris (1977) argued that an interactive PMS is a double-loop learning system. From an organizational design perspective, structures that are flexible and permeable, as well as the implementation of systems that provide timely information, are required for organizations to learn effectively (McGill and Slocum, 1993). When building a learning organization, five essential characteristics are necessary, including clarity of mission, leadership commitment/empowerment, experimentation/rewards, transfer of knowledge, and group problem solving (Goh and Richards, 1997). These facilitating factors can be achieved through interactive use of PMS. This is again mentioned by most researchers who have conducted an empirical analysis on the subject. For instance, according to Henri (2006) and Budianto and Yuliansyah (2014), the interactive use of PMS is positively associated with organizational learning. Senior managers promote the interactive use of PMS to stimulate organizational learning and encourage new strategies (Simons, 1995). Additionally, a PMS fundamentally influences the chance for the alteration of organizational practices (Fried, 2010). Considering the above argument, we assume that interactive use of PMS can promote organizational learning and offer the following assumption:

*Hypothesis 1:* Interactive PMS positively influences organizational learning.

### Organizational Learning and Job Performance

Based on organizational theory, there is a positive relationship between control systems and performance. Additionally, organizational performance has been significantly associated with an increased use of non-financial performance measures (Baines and Langfield-Smith, 2003). Groen et al. (2017) suggested that employee job performance was only higher when performance metrics were used for periodic discussions and evaluation purposes. Budianto and Yuliansyah (2014) found that interactive use of PMS has a direct effect on corporation performance. In order words, PMS use may encourage employees to be proactive in the attainment of superior organizational performance. However, theoretical evidence is still insufficient to suggest a direct relationship between PMS and performance at an organizational level (Abernethy and Brownell, 1999; Bisbe and Otley, 2004). Therefore, in order to contribute to the body of empirical evidence, the current study examined the use of interactive PMS to determine if it directly affects job performance.

The interactive use of PMS includes a wide range of information, providing essential indicators of threats and opportunities and ultimately seeking to enhance employee performance. Thus, we propose the following.

*Hypothesis 2:* Organizational learning positively influences job performance.

### The Mediating Role of Organizational Learning

According to knowledge theory, organizational learning is crucial for survival and growth in times of high uncertainty and variability, as it can facilitate the creation of a competitive advantage and, ultimately, superior performance (Hunt and Morgan, 1995; Slater and Narver, 1998; Mu and Di Benedetto, 2011). Recent literature (e.g., Koufteros et al., 2014) supports the notion that the interactive use of PMS advances organizational capabilities, which subsequently helps the organization meet its targets. Other studies (Simons, 1995; Henri, 2006) have emphasized that the interactive use of PMS stimulates organizational learning. However, till date, this has not been widely tested in empirical studies. Previous studies have not necessarily suggested that the use of PMS is an antecedent to organizational learning. Further, any links between the interactive use of PMS and organizational learning, and whether these can help determine employee job performance have not been examined. Henri (2006) suggested that the interactive use of PMS has a tangible impact on organizational learning. Moreover, the same positive significant relationships have also been suggested by the robustness of the theoretical model using environmental uncertainty, size, and organizational culture as splitting variables. Mu and Di Benedetto (2011) argued that organizational learning plays a pivotal role in the relationship between strategic orientation and performance. Interactive PMS may affect organizational learning by forming a positive learning atmosphere to motivate participants in terms of knowledge integration, knowledge replication, and knowledge distribution. Hence, when PMS is used interactively, it may influence employee job performance through its impact on organizational learning and meeting organizational targets. Therefore, we propose organizational learning as an explanatory mechanism in the relationship between interactive use of PMS and job performance.

*Hypothesis 3:* Interactive use of PMS exerts an indirect effect on job performance through its contribution to organizational learning.

## Materials and Methods

### Participants and Procedure

The research design enabled the researchers to target respondents who possess substantive organizational-level knowledge and specific knowledge, as it pertains to management tools. We targeted high-level executives [i.e., chief executive officer (CEO)/general manager and senior vice presidents] as well as controllers and managers (e.g., department manager and team manager). Master of business administration (MBA) students from Ningbo University and accounting leading personnel of Ningbo city were invited to participate in the study. Participation was voluntary. Besides, all surveys are anonymous, and we promised participants that all their information will be kept confidential and will only be used for research. To assure that the questions could be correctly understood by respondents and easily answered by them, the initial survey questionnaire was carefully pretested.

Data were collected through a web-based questionnaire (wjx.cn) and an on-site survey. A total of 260 questionnaires were sent, and 198 were returned. After exclusion of questionnaires with incomplete information and fuzzy information, 191 valid responses were analyzed (response rate of 73.46%). In organizational sciences literature, a few pieces of evidence are available regarding response rate. Baruch and Holtom (2008) recommended that a 60% response rate is an acceptable figure. Thus, the response rate of the present study is acceptable and allows the authors to proceed for a data analysis (see Baruch and Holtom, 2008). In order to identify whether early respondents differed from late respondents, the two groups across the mean score of each variable were compared. A Student’s *t*-test found no significant differences in terms of the interactive use of PMS, organization learning, or job performance. These results add additional support to the quality of the data and subsequent findings of the present study.

### Measures

All English-based measures were translated into Chinese according to the “translation/back-translation” procedures. Respondents rated all items on a seven-point fully anchored Likert scale (1 = *strongly disagree*, 4 = *neutral*, and 7 = *strongly agree*).

#### Interactive Use of Performance Measurement Systems

Interactive use of PMS refers to the extent to which top management teams use performance measures to build internal pressure to break out of narrow search routines, stimulate opportunity seeking, and encourage the emergence of new strategic initiatives (Simons, 1995). It was measured using an adapted version of Henri’s (2006) and Widener’s (2007) instrument. Five indicators were measured: (i) enable discussion in meetings of superiors, subordinates, and peers; (ii) enable continual challenge and debate of underlying data, assumptions, and action plans; (iii) provide a common view of the organization; (iv) enable the organization to focus on critical success factors; and (v) develop a common vocabulary in the organization. Cronbach’s alpha reliability for interactive use of PMS was adequate (Cronbach’s α = 0.906).

#### Organizational Learning

Organizational learning was measured via the scale developed by Fiol and Lyles (1985). Four indicators were examined: (i) belief that the ability to learn is the key to improvement; (ii) basic values include learning as a key to improvement; (iii) belief that once we quit learning, we endanger our future; and (iv) belief that employee learning is an investment, not an expense. Cronbach’s alpha reliability for organizational learning was adequate (Cronbach’s α = 0.871).

#### Job Performance

Job performance refers to employees’ self-evaluation of performance and the extent to which their work performance was successfully executed. We operationalized job performance via a nine-item scale, which drew items from Mahoney et al. (1965). The measurement consisted of the following indicators: (i) determining goals, policies, and courses of action; (ii) collecting and preparing information, usually in the form of records, reports, and accounts; (iii) exchanging information with individuals in the organization other than subordinates; (iv) assessment and appraisal of proposals or of reported or observed performance; (v) directing, leading, and developing subordinates; (vi) maintaining the workforce of a unit or of several units; (vii) purchasing, selling, or contracting for goods or services; (viii) advancing general organizational interests through speeches, consultation, and contacts with individuals or groups outside the organization; and (ix) overall performance. Cronbach’s alpha reliability for job performance was adequate (Cronbach’s α = 0.936).

#### Control Variables

In order to check the significant difference across an outcome variable, one-way ANOVA was performed on the collected data. As per the results, the authors found an insignificant difference across number of employees (*F* = 1.09; *p* > 0.05) and status (*F* = 1.55; *p* > 0.05) and an significant difference across position (*F* = 6.04; *p* < 0.05), gender (*F* = 5.18; *p* < 0.05), and age (*F* = 5.75; *p* < 0.05). Hence, position, gender, and age were included as control variables in our study.

#### Analytical Strategy

Bootstrapping was used to test a structural equation model via SAS9.4 and Smart-PLS3.0. Partial least squares (PLS) has become more and more popular in recent years owing to its specific advantages, such as minimal requirements on measurement scales and sample distribution (Qureshi and Compeau, 2009; Guo and Barnes, 2011; Cäker and Siverbo, 2018). First, a principal component analysis was conducted to extract factors for the exploratory factor analysis (EFA). Then, convergent validity and discriminant validity were tested, as well as internal consistency/reliability of latent variables via a confirmatory factor analysis (CFA). The Fornell–Larcker criterion, which stipulates that each latent construct should have a higher average variance extracted (AVE) than the highest squared correlation with any other construct, was satisfactorily met for all main constructs. Additionally, all indicators loaded the highest on their own scales. This means the measurement model displayed good discriminant validity. Furthermore, to test the proposed relationships, PLS–structural equation model path coefficients were examined.

## Results

### Demographics

The demographics appear in Table 1. Among a total of 191 managers, 54.97% are male and 45.03% are female. For age, 28.80% are under 30 years old, 22.51% are 31–35 years old, 37.17% are 36–40 years old, 9.95% are 41–45 years old, and 1.57% are aged more than 46 years. For position, 4.71% are CEO/general manager, 10.99% are senior vice presidents, 45.03% are department manager, and 39.27% are team manager. Around 24.08% of the firms are considered small, with employment levels below 100, whereas 27.75% of the firms have a size greater than 2,000. In the case of status of the company, 32.46% are public, 32.46% are private, 16.23% are wholly foreign owned, 5.76% are joint ventures, and 13.09% are other. Moreover, 93.72% of the companies are located in Zhejiang Province.

**TABLE 1:** Demographics.

Items	Classification	Frequency	Percentage
Gender	Male	105	54.97
	Female	86	45.03
Age category	Under 30 years	55	28.80
	Between 31 and 35 years	43	22.51
	Between 36 and 40 years	71	37.17
	Between 41 and 45 years	19	9.95
	More than (and equal to) 46 years	3	1.57
Number of employees	Fewer than 100	46	24.08
	Between 101 and 500	45	23.56
	Between 501 and 2,000	47	24.61
	Between 2001 and 5,000	15	7.85
	More than (and equal to) 5,001	38	19.90
Location of the company	Beijing	4	2.09
	Shanghai	4	2.09
	Zhejiang (Province)	179	93.72
	Jiangsu (Province)	4	2.09
Status of the company	Public	62	32.46
	Private	62	32.46
	Wholly foreign owned	31	16.23
	Joint ventures	11	5.76
	Other	25	13.09
Position	Team manager	75	39.27
	Department manager	86	45.03
	Senior vice presidents	21	10.99
	CEO/general manager	9	4.71

*CEO, chief executive officer.*

### Exploratory Factor Analysis

Exploratory factor analysis revealed factor loadings with values ranging from 0.720 to 0.852, with three factors exhibiting eigenvalues > 1.0, explaining 69.972% of the total variance (see Table 2). Descriptive statistics found a mean PMS use score of 5.013, with item 4 demonstrating a maximum mean of 5.136, indicating frequent use of this item within the sample. Further, these results suggest high importance of interactive PMS use. Organizational learning item 4 demonstrated a maximum mean of 5.042. Further, a mean of 4.934 was observed for job performance, with little difference between each item.

**TABLE 2:** Exploratory factor analysis and descriptive statistics.

Constructs	Items	Factor 1	Factor 2	Factor 3	Mean ± SD
Interactive use of PMS	pms1	**0.850**	0.013	0.087	4.995 ± 1.254
	pms2	**0.838**	0.172	0.037	4.953 ± 1.266
	pms3	**0.840**	0.187	0.159	5.052 ± 1.284
	pms4	**0.852**	0.166	0.180	5.136 ± 1.319
	pms5	**0.773**	0.194	0.144	4.927 ± 1.445
	Interactive use of PMS				5.013 ± 1.120
Organizational learning	cap21	0.180	**0.780**	0.276	4.948 ± 1.297
	cap22	0.099	**0.824**	0.319	4.890 ± 1.351
	cap23	0.176	**0.851**	0.092	4.853 ± 1.372
	cap24	0.217	**0.755**	0.160	5.042 ± 1.353
	Organizational learning				4.933 ± 1.140
Job performance	jp1	0.124	0.203	**0.792**	4.859 ± 1.093
	jp2	0.158	0.147	**0.816**	5.047 ± 0.980
	jp3	0.104	0.141	**0.814**	4.838 ± 0.984
	jp4	0.073	0.228	**0.766**	4.832 ± 1.063
	jp5	0.054	0.128	**0.820**	4.874 ± 0.987
	jp6	0.042	0.216	**0.720**	4.848 ± 1.068
	jp7	0.132	0.107	**0.786**	4.953 ± 1.053
	jp8	0.138	0.051	**0.785**	5.042 ± 1.004
	jp9	0.123	0.142	**0.835**	5.110 ± 1.002
	Job performance				4.934 ± 0.835
Eigenvalue		3.688	2.943	5.964	
Percentage of variance explained (%)		20.489	16.350	33.133	
Total variance (%)		20.489	36.839	69.972	

*“pms”, “cap,” and “jp” represent interactive use of PMS, organizational learning, and job performance, respectively. PMS, performance measurement system. The data in bold represent the loading of an item.*

### Confirmatory Factor Analysis

Additionally, a CFA showed that all item-factor loadings were greater than 0.60, with values in the 0.754–0.901 range, suggesting good convergent validity (see Table 3). Table 4 presents validity and reliability statistics. Composite reliability was well above 0.7, and AVE was well above 0.5 for all constructs. Further, all Cronbach’s alpha values were above 0.6, indicating good internal consistency (Yoo and Alavi, 2001). Additionally, PLS correlations and squared AVEs (see Table 5) showed that the measurement model displayed good discriminant validity.

**TABLE 3:** Confirmatory factor analysis in PLS.

	Interactive use of PMS	Organizational learning	Job performance
pms1	**0.806**	0.219	0.198
pms2	**0.826**	0.338	0.183
pms3	**0.887**	0.376	0.298
pms4	**0.901**	0.366	0.314
pms5	**0.837**	0.350	0.278
cap21	0.358	**0.864**	0.432
cap22	0.296	**0.896**	0.470
cap23	0.334	**0.839**	0.282
cap24	0.360	**0.795**	0.331
jp1	0.278	0.411	**0.832**
jp2	0.294	0.383	**0.847**
jp3	0.258	0.352	**0.833**
jp4	0.245	0.403	**0.805**
jp5	0.203	0.336	**0.827**
jp6	0.197	0.373	**0.754**
jp7	0.246	0.358	**0.795**
jp8	0.241	0.311	**0.780**
jp9	0.268	0.379	**0.855**

*“pms”, “cap,” and “jp” represent interactive use of PMS, organizational learning, and job performance, respectively. PLS, partial least squares; PMS, performance measurement system. The data in bold are the loading of an item. For validity of the discriminant, the loading should be stronger than their cross-loadings on other constructs.*

**TABLE 4:** Goodness of fit of suggested model in PLS.

Constructs	Average variance extracted	Composite reliability	*R*^2^	Cronbach’s α	Communality
Interactive use of PMS	0.726	0.930	–	0.906	0.726
Organizational learning	0.721	0.912	0.157	0.871	0.721
Job performance	0.664	0.947	0.225	0.937	0.664
Global of fit of suggested model	0.299				

*PLS, partial least squares; PMS, performance measurement system.*

**TABLE 5:** Partial least squares correlations results.

	1	2	3
(1) Interactive use of PMS	**0.852**		
(2) Organizational learning	0.396	**0.849**	
(3) Job performance	0.306	0.454	**0.815**

*Diagonal elements (in bold) represent the square root of the average variance extracted (AVE). PLS, partial least squares; PMS, performance measurement system.*

### Hypothesis Testing

The mediation of organizational learning was tested in the manner recommended by Baron and Kenny (1986). In this model, the independent variable was denoted as the interactive use of PMS, the mediator was organizational learning, and the dependent variable was job performance. The relationship between the independent and dependent variables was assessed using a regression analysis. Table 6 shows that the interactive use of PMS displays a statistically significant positive association with job performance (β = 0.222, *p* < 0.0001).

**TABLE 6:** Regression analysis results.

Dependent variable	Independent variable	Regression coefficient	SD	*t*-value	*p*-value
Job performance	Interactive use of PMS	0.222	0.052	4.29	0.0001
Organizational learning	Interactive use of PMS	0.396	0.068	5.81	0.0001
Job performance	Organizational learning	0.325	0.048	6.81	0.0001

*PMS, performance measurement system.*

**FIGURE 1:**
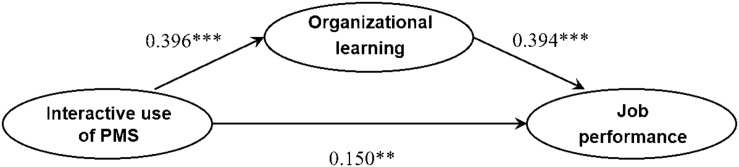
Final structural model. Paths of the control variables are omitted for clarity. Significant relationships are shown in bold. ^∗∗∗^*p* < 0.01, ^∗∗^*p* < 0.05.

A regression analysis confirmed a significant relationship between the interactive use of PMS and job performance. Therefore, in order to examine the mediating effect of organizational learning, hypotheses were tested using the PLS–path coefficient. The structural model fit was evaluated using the Wetzels et al. (2009) calculation of goodness of fit. Specifically, they derive three goodness-of-fit criteria for small (0.1∼0.25), medium (0.25∼0.36), and large (more than 0.36). Goodness of fit is defined as the geometric mean of the average communality and average *R*-square (for endogenous constructs). For the complete model, global fit was 0.299, indicating that this model was an acceptable fit to the collected data (see Table 4). The final structural model is summarized in Figure 1.

The relationship between the interactive use of PMS and organizational learning (H1) demonstrated a two-tailed significance of *p* < 0.01 (β = 0.396, *t* = 6.141). The relationship between the interactive use of PMS and job performance (H2) demonstrated two-tailed significance of *p* < 0.05 (β = 0.150, *t* = 2.048). To address the question of whether organizational learning has a mediating effect between the interactive use of PMS and job performance (H3), a significant relationship was found between the interactive use of PMS and organizational learning (H1), and also organizational learning and job performance (β = 0.394, *t* = 5.191), providing evidence in favor of a significant and positive mediating effect of organizational learning on the relationship between the interactive use of PMS and job performance.

## Discussion

The primary goal of this research was to determine how the interactive use of PMS affects employee job performance. This was accomplished by analyzing 191 questionnaires completed by individuals from Chinese firms. We investigated the mediating effect of organizational learning between the interactive use of PMS and job performance. Results show significant direct effects between the interactive use of PMS and organizational learning, and between the interactive use of PMS and job performance. In addition, organizational learning was found to mediate the relationship between the interactive use of PMS and job performance.

Results (H1) show that firms communicate with employees face to face through the interactive use of PMS, and then they create a good knowledge sharing atmosphere to improve organizational learning. Interactive PMS is characterized by focusing on constantly changing information. The interactive use of PMS is a catalyst for analyzing data, the development of action plans, and promotes face-to-face communication between superiors and subordinates, ultimately improving organizational learning among organizational members of different hierarchical levels. In other words, enterprises tend to use interactive PMS in order to improve organizational learning. Taken together, the evidence supports Henri’s (2006) claim that the interactive use of PMS is a positive force, facilitating organizational learning. The results of the current study are also complementary to the empirical studies conducted by Budianto and Yuliansyah (2014) that support the role of the interactive use of PMS in turbulent contexts.

Furthermore, results (H2) also show that the interactive use of PMS facilitates information sharing, promoting cooperation and improving communication, enabling members of the organization to actively perform tasks and improve job performance. These findings are in accordance with the previous research of Groen et al. (2017), which found that the interactive use of PMS influences job performance by facilitating periodic discussions throughout the organization.

The current study also notes organizational learning as a mediator between the interactive use of PMS and job performance. A PMS has an indirect positive effect on job performance, via organizational learning. The interactive use of PMS requires frequent and regular attention from managers at all levels of the organization, as well as an understanding of the underlying work environment to provide an open context. Additionally, PMSs play a positive role in improving organizational learning, ultimately improving job performance. An important feature of PMS is emphasizing effective communication throughout the organization. Because of its interactive use, information sharing and dialog can be carried out within the organization, which can help enhance organizational learning. As previous literature has stated, organizational learning reflects an organization’s complex capability to develop new knowledge and insights that lead to improved performance (Huber, 1991; Sinkula, 1994; Mu and Di Benedetto, 2011). Organizational learning is recognized as a primary asset necessary to achieve a competitive advantage (Cäker and Siverbo, 2018). If an organization wishes to promote learning, then interactive PMS should be considered when designing control systems.

### Theoretical Contributions and Managerial Implications

Theoretically speaking, as a first contribution, the finding that the interactive use of PMS indirectly affects job performance via organizational learning (H3) expands what is known about how to develop valid and meaningful performance management systems, and it supplements the existing research on PMSs. In the context of increasing competitiveness, this finding is helpful for managers so that they may correct their behavior through PMSs. Further, the current results are helpful to establish effective control and increase employee participation. The second contribution of the study relates to the investigation of the effects of interactive PMS on organizational learning as predicted by resource-based theory. This paper enriches the literature examining a PMS and organizational learning by providing practical evidence for organizations on the basis of a questionnaire data analysis.

In practical terms, the results of the current study suggest that managers should rely on employee initiative to optimize sustainability when facing a highly competitive environment. The acquisition of sustained competitiveness is the result of sharing ideas. Improved performance can only be achieved through face-to-face communication at all levels of the organization. Information generated by a PMS concerns ongoing changes with potential opportunities, such as changes in technology, consumer preferences, governmental regulations, and industry competition. Therefore, in order to improve the quality of organizational learning, managers should develop a common vocabulary through shared and open interactive controls.

In addition, organizational learning is the main factor that directly affects the job performance. For organizational learning to ensure improvement in job performance, employees must continuously acquire knowledge, gather new skills, and improve their behaviors. The overall results show that an increase in a PMS score leads to an increase in organizational learning and finally to an increase in employee job performance. This also suggests that managers not only should frequently pay attention to the changing information provided by the PMS but also should provide employees an environment that stimulates their productivity. Interactive PMS focuses organizational attention on stimulating dialog throughout all levels of management, motivating employees to improve their knowledge base, leading to an overall improvement in job performance.

### Study Limitations, Strengths, and Future Research Directions

Although this study sheds some light on the role of interactive PMS, several limitations should be considered. First, the current study uses a limited sample size. Data were collected in an economically advanced region of China. To increase the applicability of current results, other areas of China or elsewhere could be covered in future studies. A cross-sectional design was used in this study. Thus, although the underlying theory behind our model assumes the investigated relationships, causality cannot be concluded before it has been tested with an experimental or longitudinal design. In addition, in terms of research methodology, the results may have some subjective bias owing to the limitations of the questionnaire itself. Finally, the measurement scale of organizational learning is taken from a holistic perspective, despite the fact that organizational learning can be divided into single-loop learning and double-loop learning (Simons, 1995). Therefore, the relationship between the components of organizational learning and interactive PMS use should be further clarified. Moreover, more research could be based on a more comprehensive concept, such as environmental uncertainty and organizational culture, to explore the influence of interactive controls on employees’ job performance.

Despite having the abovementioned limitations, the current study has a number of strengths. First, the current study is a significant addition to the existing body of interactive PMS use literature. The practices of interactive PMSs are very important and observable everywhere in China, but there are a few empirical pieces of evidence in the literature. Second, this study enhances the theoretical base of the relation organizational learning and job performance by considering empirical evidences of previous research.

## Conclusion

We have explored the impact of PMSs on organizational learning and job performance. On the basis of survey responses from 191 managers in Chinese organizations, we conclude that (1) an increase in organizational learning occurs if interactive PMS is used frequently; (2) the interactive use of PMS has a significant positive association with employee job performance; and (3) the interactive use of PMS has a positive indirect effect on employee job performance, which is mediated by organizational learning.

## Data Availability Statement

The datasets for this article are not publicly available because authors have made a commitment to the participants to not share their answers/data. Requests to access the datasets should be directed to zhanglu1@nbu.edu.cn.

## Ethics Statement

Ethical review and approval was not required for the study on human participants in accordance with the local legislation and institutional requirements. The patients/participants provided their written informed consent to participate in this study.

## Author Contributions

LZ: conceptualization, methodology, software, formal analysis, writing, and original draft preparation. WY: writing, reviewing, and editing of the manuscript, visualization, supervision, project administration, and funding acquisition.

## Conflict of Interest

The authors declare that the research was conducted in the absence of any commercial or financial relationships that could be construed as a potential conflict of interest.
